# Characterization and modeling of the *Haemophilus influenzae *core and supragenomes based on the complete genomic sequences of Rd and 12 clinical nontypeable strains

**DOI:** 10.1186/gb-2007-8-6-r103

**Published:** 2007-06-05

**Authors:** Justin S Hogg, Fen Z Hu, Benjamin Janto, Robert Boissy, Jay Hayes, Randy Keefe, J Christopher Post, Garth D Ehrlich

**Affiliations:** 1 Allegheny General Hospital, Allegheny-Singer Research Institute, Center for Genomic Sciences, Pittsburgh, Pennsylvania 15212, USA; 2 Joint Carnegie Mellon University - University of Pittsburgh Ph.D. Program in Computational Biology. 3064 Biomedical Science Tower 3, 3501 Fifth Avenue, Pittsburgh, Pennsylvania 15260, USA

## Abstract

**Background:**

The distributed genome hypothesis (DGH) posits that chronic bacterial pathogens utilize polyclonal infection and reassortment of genic characters to ensure persistence in the face of adaptive host defenses. Studies based on random sequencing of multiple strain libraries suggested that free-living bacterial species possess a supragenome that is much larger than the genome of any single bacterium.

**Results:**

We derived high depth genomic coverage of nine nontypeable *Haemophilus influenzae *(NTHi) clinical isolates, bringing to 13 the number of sequenced NTHi genomes. Clustering identified 2,786 genes, of which 1,461 were common to all strains, with each of the remaining 1,328 found in a subset of strains; the number of clusters ranged from 1,686 to 1,878 per strain. Genic differences of between 96 and 585 were identified per strain pair. Comparisons of each of the NTHi strains with the Rd strain revealed between 107 and 158 insertions and 100 and 213 deletions per genome. The mean insertion and deletion sizes were 1,356 and 1,020 base-pairs, respectively, with mean maximum insertions and deletions of 26,977 and 37,299 base-pairs. This relatively large number of small rearrangements among strains is in keeping with what is known about the transformation mechanisms in this naturally competent pathogen.

**Conclusion:**

A finite supragenome model was developed to explain the distribution of genes among strains. The model predicts that the NTHi supragenome contains between 4,425 and 6,052 genes with most uncertainty regarding the number of rare genes, those that have a frequency of <0.1 among strains; collectively, these results support the DGH.

## Abstract 2

The genomes of 9 non-typeable *H. influenzae *clinical isolates were sequenced and compared with a reference strain, allowing the characterisation and modelling of the core-and supra genomes of this organism.

## Background

*Haemophilus influenzae *is a Gram-negative bacterium that colonizes the human nasopharynx and is also etiologically associated with a spectrum of acute and chronic diseases. There are six recognized capsular serotypes (a-f), but the majority of clinical strains are unencapsulated and are referred to as nontypeable *H. influenzae *(NTHi). The type b polysaccharide capsular variants (Hib) are associated with invasive disease, particularly meningitis; however, the introduction of a highly effective vaccine has nearly eliminated this pathogen from developed countries. Recent studies have demonstrated that the NTHi form biofilms on the respiratory mucosa of humans and other mammals and it has been hypothesized that this contributes to the chronicity of these infections [1,2]. They are the most frequently detected pathogens associated with both the acute and chronic forms of otitis media (OM) [3] and also are recognized as a seed pathogen in a wide range of chronic polymicrobial infections of the respiratory mucosa, including the cystic fibrosis lung, chronic obstructive pulmonary disease, tracheobronchitis, rhinosinusitis, and mastoiditis [4,5].

The NTHi are naturally transformable and their genomes demonstrate a high degree of plasticity among strains [4,6-11]. Previous work from our laboratory has shown that approximately 10% of the genes possessed by each clinically isolated strain are novel with respect to the reference strain Rd KW20 and that the distribution of these genes among the strains is non-uniform [11]. Polyclonal NTHi populations have been associated with chronic disease as well as with nasopharyngeal carriage [4,12], while other researchers have observed *in situ *horizontal gene transfer in diseased patients [7,8,13]. The twin observations that the NTHi form biofilms during chronic infections and that these infections are often polyclonal suggests that multiple unique strains are co-localized within an environment demonstrated to support greatly elevated rates of horizontal gene transfer [14-18]. These circumstantial evidences suggest that a genetically diverse population may be important to the fitness of *H. influenzae *as a human pathogen and that continuous horizontal gene transfer among co-colonizing strains is the mechanism that generates the diversity observed in the population. It has been hypothesized that this microbial diversity generation is the counterpoint to the adaptive immune response of the mammalian host [19]. The distributed genome hypothesis (DGH) states that the full complement of genes available to a pathogenic bacterial species exists in a 'supragenome' pool that is not contained by any particular strain, but is available through a genically diverse population of naturally transformable bacterial strains. The distributed genome is not a phenomenon isolated to *H. influenzae*; comparative genomic studies in other bacterial pathogens, including pneumococcus and *Pseudomonas aeruginosa*, have demonstrated even greater degrees of genomic plasticity among clinical strains [20,21]. Moreover, evolutionary studies have demonstrated that pneumococcus uses competence and transformation as a pathogenic mechanism [22-24].

Testing of the DGH and its predictions will provide insight into clinically relevant problems, such as antibiotic resistance, chronic biofilm disease, and serotype-diverse species, which readily adapt to standard vaccinations. Further characterization of the *H. influenzae *supragenome is a prerequisite to addressing these issues. In this regard we have sequenced the genomes of 11 clinical NTHi isolates, 2 by standard clone-based Sanger sequencing and 9 using the new 454-based pyrosequencing technology. This dataset, combined with the published genomic sequences of Rd and R2866, constitutes the largest set of genomic data collected for *H. influenzae *to date - the first step towards a characterization of the full complement of genes that collectively define the *H. influenzae *supragenome. In this paper we present a global comparative analysis that characterizes the distribution of genetic diversity among the strains.

## Results

### DNA sequence data

Table 1 lists the 12 *H. influenzae *clinical strains and the reference strain Rd, a largely non-pathogenic strain, used in the comparative genomic studies described herein, their NCBI locus tags, the location where the sequencing was performed, and their clinical origins. Nine of the clinical strains were sequenced using 454 LifeSciences novel pyrosequencing technology [25]. The number of sequencing runs, the extent of genomic coverage, and the number of contigs resulting from first and in some cases second pass assemblies are tabulated (Table 2).

**Table 1:** Bacterial strains and sources used for whole genome sequencing, comparative genomics, and computation of the NTHi core and supragenomes

NTHi strain	NCBI locus tag prefix	Sequence source	Clinical source [reference]
Rd KW20	HI	NCBI	Lab strain, formerly serotype D [32]
86-028NP	NTHI	NCBI	NP isolate from COM patient [33]
R2846	N/A	SBRI	OM isolate, St Louis [10,52]
R2866	N/A	SBRI	Blood isolate (meningitis), Seattle [10,53]
3655	CGSHi3655	CGS	AOM isolate, Missouri [54, from A. Ryan]
PittAA	CGSHiAA	CGS	OME isolate, Pittsburgh [11]
PittEE	CGSHiEE	CGS	OME isolate, Pittsburgh [11]
PittGG	CGSHiGG	CGS	Otorrhea isolate, Pittsburgh [11]
PittHH	CGSHiHH	CGS	OME isolate, Pittsburgh [11]
PittII	CGSHiII	CGS	Otorrhea isolate, Pittsburgh [11]
R3021	CGSHiR3021	CGS	NP isolate [10]
22.4-21	CGSHi22421	CGS	NP isolate, Michigan [12]*
22.1-21	CGSHi22121	CGS	NP isolate, Michigan [12]*

AOM, acute otitis media; CGS, Center for Genomic Sciences; NP, nasopharyngeal; N/A, not available; OM, otitis media; OME, otitis media with effusion; SBRI, Seattle Biomedical Research Institute.

**Table 2:** Sequencing data for the 9 Nthi strains sequenced with 454-technology

*H. influenzae *strain	40×70 plates sequenced	454 read coverage	No. of Newbler contigs	PCR gap closure?	4 kb clone library?	Final no. of contigs
3655	2	30×	59	No	No	59
PittAA	1	23×	88	Yes	No	38
PittEE	2	42×	49	Yes	4× cover	12
PittGG	1	21×	60	No	Yes*	60
PittHH	2	48×	73	No	No	73
PittII	1	16×	205	No	Yes	205
22.4-21	1	19×	69	No	No	69
R3021	2	35×	51	No	No	51
22.1-21	1	19×	71	No	No	71

*Clone library not incorporated in present analysis.

### Determination of gene clustering parameters

**Figure 1:**
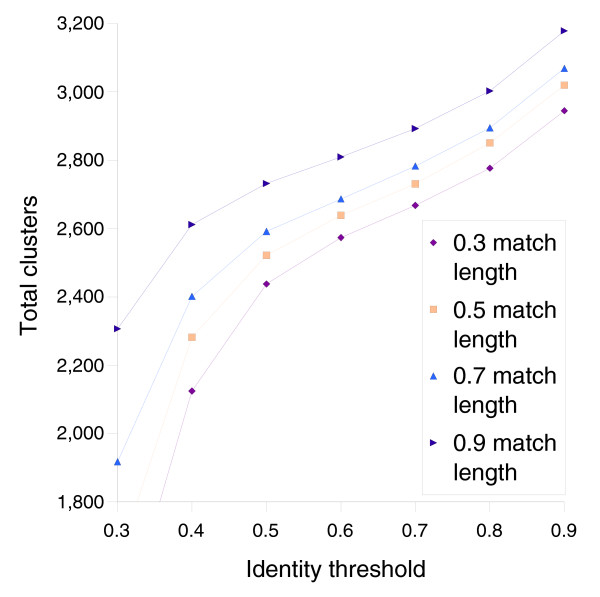
A plot of the total number of clusters as a function of clustering parameters shows an inflection point near 0.65 identity and 0.70 match length. The inflection, which minimizes the rate of change in the number of clusters per change in parameters, suggests a set of parameters that optimally segregates orthologs and paralogs.

Gene clustering parameters for the grouping of homologs were empirically determined by minimizing the change in the number of clusters per change in the parameters (Figure 1). We hypothesize that this minimum point coincides with the best estimate threshold for distinguishing true orthologs from functionally distinct homologs. Some homologs will be more similar than 70%, while some orthologs will be more divergent than 70%, but as a uniform criterion, the threshold is optimized. Visual inspection of the clusters reveals that most clusters are reasonable. Mosaic genes were particularly difficult to cluster due to high levels of rearrangement. In the remainder of the paper, genes in the same cluster are considered to be the same gene.

### Enumeration of gene clusters and genic relationships among NTHi strains

**Figure 2:**
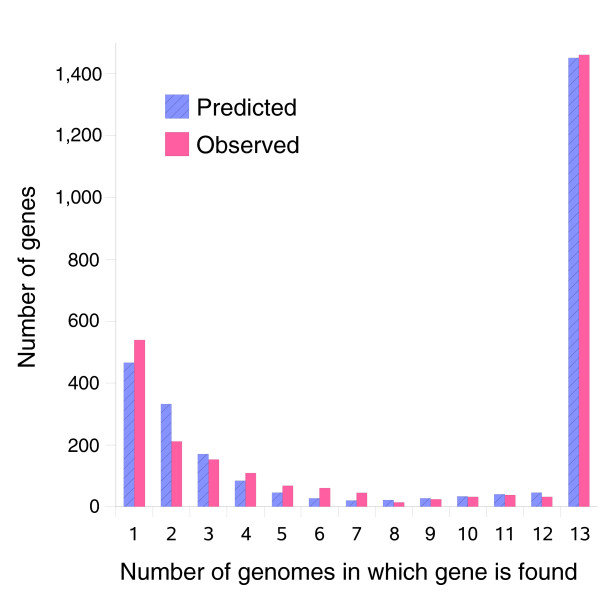
A histogram of gene clusters observed in exactly *N *of 13 *H. influenzae *strains compared to the expected number of genes estimated by the supragenome model (trained on all 13 strains). Over 1,400 genes were observed in all 13 strains, indicating that there is a common core set of genes. Distributed genes appear in variable numbers of strains, from 1 to 12. Overall, the model fits the data well, though it underestimated the number of genes observed once and overestimated the number of genes observed twice.

**Figure 3:**
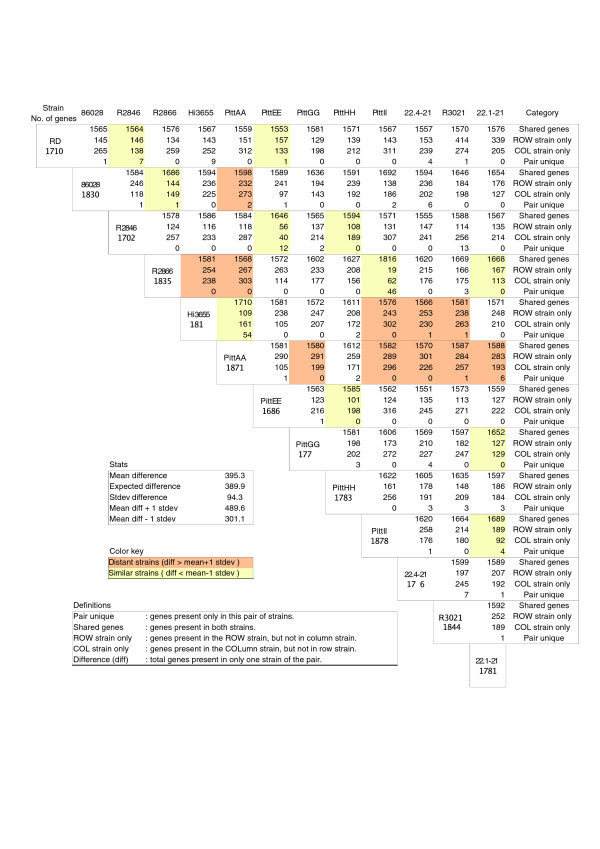
A pairwise genic comparison of 12 NTHi strains of *H. influenzae *and the reference strain Rd KW20. The comparison of two strains is found at the intersection of the row and column corresponding to the respective strains. Strains are compared based on the number of genes shared between the pair, the number of genes found in one strain but not the other, and the number of shared genes that are unique to that pair of strains. A typical pair of strains differs by 395 genes. Similar pairs of strains are shaded in yellow, while divergent strains are shaded orange.

We identified 2,786 gene clusters among the 13 strains (Table 3). Of these, 52% were found in every strain (core genes) and 19% were found in only a single strain (unique genes). The remaining 29% of genes were found in some combination of two or more strains, but not all (distributed genes; Figure 2). The number of clusters found per strain varied from 1,686 in PittEE to 1,878 in PittII (Table 4). All strains possessed some unique genes not seen in any of the other strains. A pair-wise comparison was performed among all possible strain pairs, which determined the mean number of genic differences between any two strains was 395 with a standard deviation of 94 (Figure 3). This analysis also identified minimal and maximal genic differences of 81 and 577, respectively, for the strain pairs 2866:PittII and 2866:PittAA. The number of coding sequences identified per genome by AMIgene did not correlate strongly with genome size. This is likely due to the presence of split open reading frames (ORFs) in the 454 sequenced genomes as an analysis of the 4 completed genomes showed a linear relationship between gene number and genome size with an R^2 ^= 0.910. In contrast, the correlation between total gene clusters and genome size is 0.86, implying that the number of distinct genes found on the genome is linearly related to the genome size.

**Table 3:** Gene clustering results

Total gene clusters	2,786
Core gene clusters	1,461
Contingency clusters	1,325
Unique clusters	539

**Table 4:** Gene identification and clustering results

*H. influenzae *strain	Genome size (MB)	No. of AMIgene CDSs found	Total gene clusters	Contingency gene clusters	Unique gene clusters
Rd KW20	1.83	1,802	1,710	271	52
86028-NP	1.91	1,867	1,830	391	28
R2846	1.82	1,729	1,702	263	4
R2866	1.93	1,864	1,835	396	1
3655	1.85	1,880	1,819	380	62
PittAA	1.92	1,971	1,871	432	98
PittEE	1.80	1,762	1,686	247	19
PittGG	1.84	2,038	1,779	340	53
PittHH	1.83	1,931	1,783	344	45
PittHII	1.92	2,245	1,878	439	26
22.4-21	1.84	2,264	1,796	357	86
R3021	1.89	2,075	1,844	405	55
22.1-21	1.85	2,181	1,781	342	10

**Figure 4:**
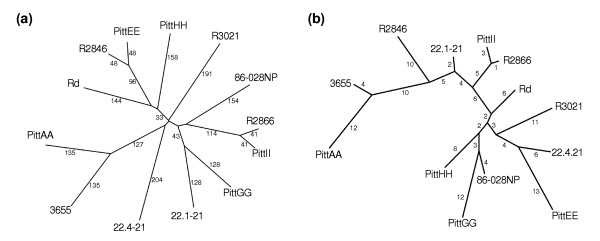
Plotting of relationships among the sequenced NTHi strains by gene sharing and multi-locus sequence typing. **(a) **A dendrogram based on genic differences among the 13 strains of *H. influenzae*. While several pairs of strains appear to be closely related, there is not a well-defined clade structure. The dendrogram was generated using the unweighted pair group method with arithmetic mean (UPGMA) method [44-46]. The number on each branch corresponds to the number of genic differences from the previous branch point. **(b) **A dendrogram based on sequence alignments of the seven MLST loci. The tree was built using the maximum likelihood method implemented in fastDNAml. The number on each branch corresponds to the number of point mutations per kilobase from the previous branch point. The topologies of the genic and MLST based trees are different. Most notably, strains PittEE and R2846 are closely related in the genic dendrogram, but are separated in the MLST dendrogram. In other instances, such as PittII and R2866, the strains are closely related in both trees.

A dendrogram based on non-core genic differences (Figure 4a) demonstrates the diversity in the NTHi population. A typical strain differs from its nearest neighbor by more than 200 genes. The strains collected from otitis media with effusion (OME) patients at Children's Hospital in Pittsburgh (designated as Pitt strains) show that a genetically diverse population can be isolated contemporaneously from a single geographic location from patients with similar indications. In contrast, two pairs of strains, PittEE/R2846 and PittII/R2866 are relatively similar despite geographically distinct points of isolation. Interestingly, the laboratory strain Rd KW20 is not an outlier among the clinical strains. For comparison, a maximum likelihood tree was generated using sequence from seven multi-locus sequence typing (MLST) housekeeping genes for the same set of 13 strains (Figure 4b). The topology of the trees is significantly different, both in terms of pairwise groupings and overall structure.

**Figure 5:**
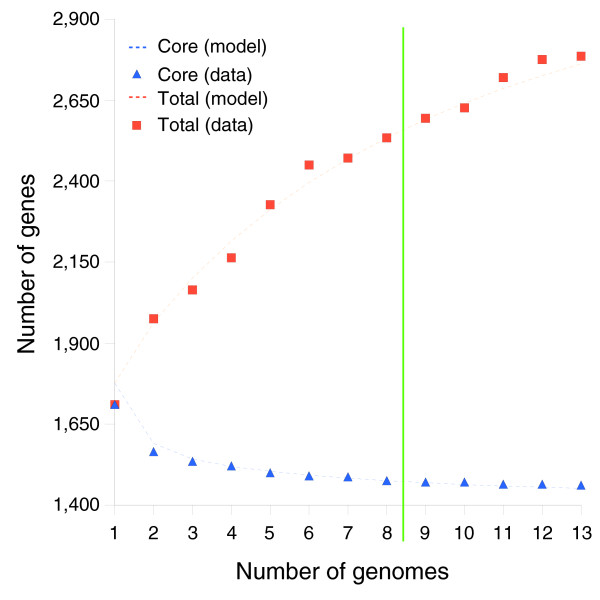
The expected number of total gene clusters and core gene clusters identified at the addition of each genome to the clustering dataset. Modeling predictions are based on the eight strain training set (see 'Mathematical development of a finite supragenome model'). The number of genes observed in all strains levels off to an asymptote that corresponds to a core set of genes. The rate of increase in total genes decreases, but does not level off due to the discovery of rare genes.

**Figure 6:**
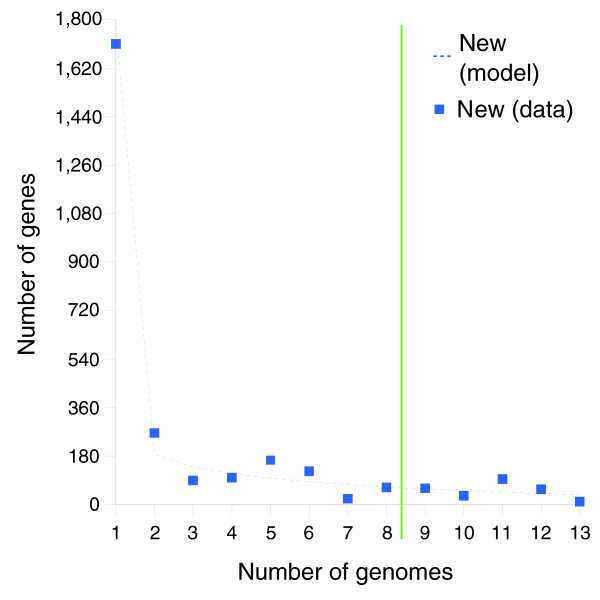
The observed and expected number of new gene clusters found at the addition of each genome to the clustering dataset. Modeling predictions are based on the eight strain training set (see 'Mathematical development of a finite supragenome model').

The identified number of new genes and core genes found per addition of each genome (as determined by incremental clustering of the 13 strains) shows an exponentially decaying trend in both cases (Figures 5 and 6). Qualitative inspection suggests a diminishing return on new genes found in future sequences, though it is expected that approximately 40 new gene clusters will be found in each of the next few genomes that are sequenced. The number of core genes appears to trend towards a horizontal asymptote near 1,450 genes. A quantitative analysis of these results is developed below in the section 'Mathematical development of a finite supragenome model'.

### Whole genome alignments reinforce the great diversity observed among gene clusters

**Figure 7:**
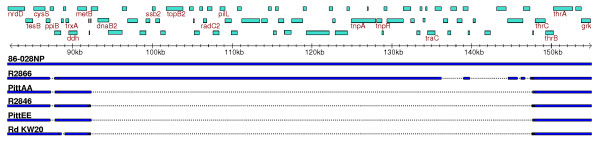
A multi-sequence alignment using 86-028NP as a reference shows varying degrees of homology among 6 strains to a 50 kb region homologous to the plasmid ICEhin1056. The plasmid is integrated in 86-028NP and is partially present in R2866, but absent from the other strains in the alignment. Sequences present in other strains without homology to 86-028NP are not shown.

**Figure 8:**
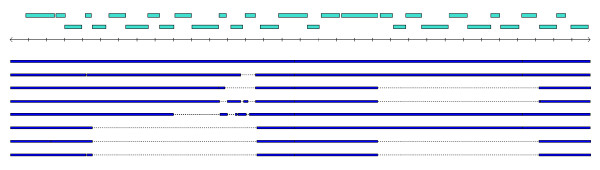
A 40 kb region present in Rd KW20 shows two blocks of genomic variation among other strains. The upstream block is bounded on the right by a frame-shifted insertion sequence (IS) element (HI1018). The downstream block (HI1024-HI1032) includes genes with likely roles in sugar transport and metabolism. Rd is used as a reference for the alignment, and sequence present in other strains without homology to Rd is not shown.

**Figure 9:**
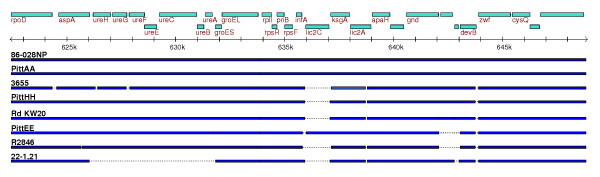
A 20 kb region that demonstrates strain diversity at the level of an individual gene (lic2C), a pair of genes (NTHi0683/4), and a group of seven functionally related genes (urease system). 86-028NP is used as a reference for the alignment, and sequence present in other strains without homology to 86-028NP is not shown.

Whole genome alignments were generated between Rd and each of the 12 clinical strains to quantify genomic insertions and deletions independently of gene identification (Table 5). On average, each of the clinical strains had 127 genomic insertions (>90 base-pairs (bp) in length) that did not correspond to any Rd KW20 sequence. Similarly, each clinical strain contained, on average, 147 genomic deletions (>90 bp) when compared to the Rd KW20 strain. The average total length of non-matching sequences between the 12 clinical strains and Rd was 321 kb, approximately 18% of the genome. The quantity of non-matching sequences reasonably accounts for the average of 390 genic differences between strain pairs. Figure 7 shows a genomic region in which two different forms of an insert, homologous to the plasmid ICEhin, have integrated into the same site of two different genomes, but which is wholly absent from the other strains in the alignment. Similarly, a 40 kb contiguous region in Rd shows extensive deletional diversity among seven of the clinical strains, with only two of the clinical strains demonstrating the same local genomic organization (Figure 8). Interestingly, the two strains, PittAA and PittEE, that are similar in this region are highly divergent overall (Figure 3). Genic diversity also exists on a smaller scale. Figure 9 displays a 20 kb region from 7 clinical strains that shows 5 different combinations of possession and loss of the lic2C gene, the NTHI0683 gene, and the UreABCEFGH operon.

**Table 5:** Analysis of inserted and deleted Sequence in 12 strains with respect to Rd KW20

Reference: Rd KW20	86-028	R2846	R2866	3655	PittAA	PittEE	PittGG	PittHH	PittII	22.4-21	22.1-21	R3021
Number of insertions	118	107	115	139	136	136	119	124	158	131	128	118
Median insert length (bp)	310	250	315	191	360	290	192	237	167	179	215	260
Mean insert length (bp)	2,076	1,199	2,041	1,248	1,245	961	1,419	1,408	879	1,274	959	1,869
Max insert length (bp)	55,275	13,119	53,044	15,789	20,222	9,796	28,306	32,587	11,085	14,983	10,810	58,706
Total insert length (bp)	244,946	128,290	234,704	173,459	169,310	130,683	168,840	174,636	138,906	166,923	122,721	220,535
Number of deletions	120	100	106	178	129	110	158	169	213	172	156	159
Median deleted length (bp)	276	268	359	274	288	264	195	205	246	317	357	340
Mean deleted length (bp)	1,254	1,354	1,128	900	1,339	1,340	816	874	708	990	898	938
Max deleted length (bp)	41,022	34,677	41,021	17,858	38,501	33,544	38,506	38,367	41,021	41,022	41,021	41,022
Total deleted length (bp)	150,491	135,377	119,612	160,262	172,723	147,451	128,936	147,689	150,857	170,262	140,021	149,079

All results are quantified with respect to Rd KW20.

**Figure 10:**
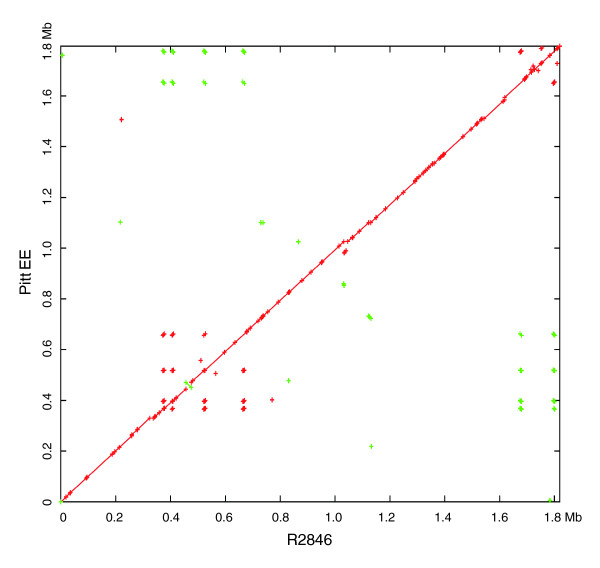
A global alignment of R2846 and PittEE as visualized by Mummerplot. A point is placed at the (x,y) coordinate if the x-coordinate of R2846 matches the y-coordinate of PittEE. Green matches indicate a reverse complement match. It can be seen that PittEE and R2846 are similar at the global level.

**Figure 11:**
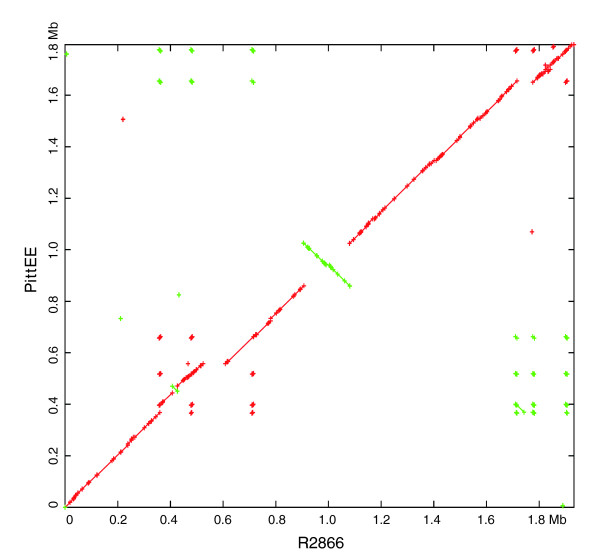
Global alignment of R2866 and PittEE shows a large inversion and several regions unique to each strain. The strains are similar across the majority of the genome; however, there is one large inversion as well as several regions unique to each strain.

Global genomic alignments of PittEE against R2846 and R2866 were performed (Figures 10 and 11). PittEE and R2846 are very similar at the global level and this is reinforced by the gene cluster analysis, which revealed only 96 genic differences. In contrast, R2866 has a large inversion and several large insertions and deletions with respect to PittEE. This diversity at the global level corresponds to the 377 genic differences identified between these two strains by cluster analysis (Figure 3). Global alignments were not visualized for most strains since the ordering of the contigs had not been determined.

### Codon usage analysis

**Figure 12:**
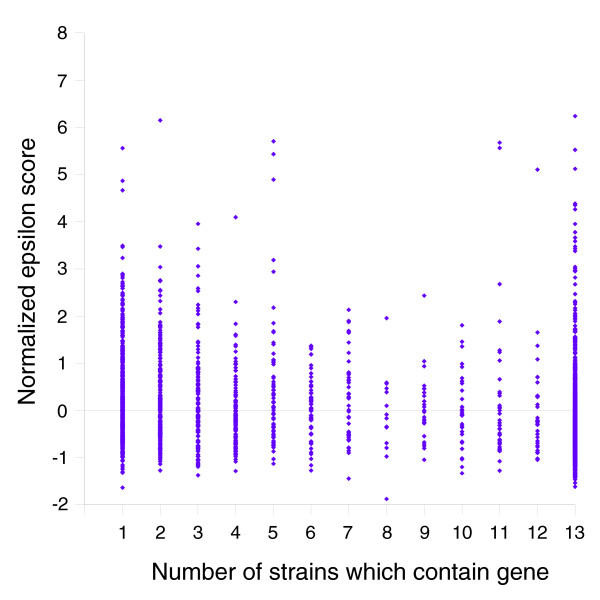
Codon usage of genes is quantified by a normalized epsilon score [26]. Low epsilon scores indicate that a gene's codon usage is similar to the typical *H. influenzae *codon usage pattern. The range of epsilon scores is similar for all three classes of genes: unique, distributed and core. However, the median scores are significantly different among the classes. The observation that the distributions for non-core genes overlap with the core genes suggests that many of the non-core genes have been evolving in the same pool with the core genes.

The codon usage of each gene cluster was compared to the typical *H. influenzae *codon usage pattern by the epsilon-score calculated by CodeSquare [26]. A low epsilon score indicates that a gene's codon usage is similar to typical patterns of the organism, while a high score indicates atypical codon usage. Since the epsilon score is partially dependent on the length of a coding sequence, all scores were normalized by length. The average normalized score is 0 and low values continue to indicate typical codon usage. Figure 12 is a scatter plot of the normalized epsilon scores versus the number of strains in which the gene was found. The range of normalized epsilon values is similar for core, distributed, and unique genes, though the median values are slightly higher for distributed and unique genes (Tables 6 and 7). The Mann Whitney U-test was employed to determine the significance of this difference. To eliminate any remaining length bias, only genes with lengths of 200-300 amino acids were analyzed. The median normalized-epsilon value of core genes is significantly smaller than the medians of distributed and unique genes, and as a consequence, these non-core genes are more likely to have foreign origins. Interestingly, there is no significant difference between distributed and unique genes and most of these non-core genes display typical *H. influenzae *codon usage.

**Table 6:** Codon usage comparisons of core, contingency and unique genes

Group 1	Group 2	*P *value
Core	Unique	5.34E-16
Core	Distributed	4.95E-16
Core	Non-core	6.55E-25
Contingency	Unique	0.17

The Mann Whitney U-test for significant differences in median of epsilon scores for each pair of gene groups. Only genes with a protein coding length of 200-300 amino acids were tested to minimize length bias. Median core epsilon scores are significantly different among the three gene groups.

**Table 7:** Codon usage comparison of core, contingency and unique genes

Group	Median epsilon	Median length (amino acids)
Core	-0.57	243
Contingency	-0.01	252
Unique	0.16	248

Median epsilon scores and protein coding length for each category of genes (includes genes of all lengths).

### Phage homology analysis

Phage insertion is a common origin of genomic diversity. The influence of phage was quantified by a homology search between all gene clusters and the NCBI NT database. A gene cluster was said to be 'phage associated' if one of the top ten significant matches was annotated as a sequence of phage origin. Overall, 9.3% of gene clusters were phage associated. The distribution of these genes is not uniform among core and non-core genes. Only 0.3% of core genes were phage associated, while 14.6% and 25.8% of distributed and unique genes, respectively, were phage associated (Table 8).

**Table 8:** Percentage of genes with probable phage origin per category

Category	Total genes	Phage derived	Percent phage
Unique genes (1 strain)	539	139	25.8%
Distributed genes (2-12 strains)	786	115	14.6%
Core genes (all strains)	1,461	4	0.3%
Totals	2,786	258	9.26%

### Development of a finite supragenome model

The comparative genomic data presented above are supportive of the DGH and reinforces the concept that, at the species level, there is an *H. influenzae *supragenome that is much larger than the genome of any single individual strain, and hence many strains must be sequenced to generate an accurate picture of the species supragenome. Among the questions we may ask about the supragenome, the most obvious is, how many strains must be sequenced to observe the entire (or nearly all) of the supragenome?. The problem is similar to determining the read coverage necessary to sequence an entire individual genome using a random shotgun library approach. Lander-Waterman statistics provide an answer in the latter case by using the assumption that reads are independently and randomly sampled from the genome with equal probability. Previously, Tettelin *et al*. [27] developed a supragenome model for *S. agalactiae *that, like Lander-Waterman statistics, is based on the assumption that contingency genes are independently sampled from the supragenome with equal probability, except in the case of rare genes, which are modeled as unique events that appear only once in the entire global population. The model requires four parameters: the number of core genes, the number of contingency genes, the probability of finding a contingency gene, and the expected number of 'unique' genes found per strain. This model predicted that the supragenome of *S. agalactiae *is infinite in size (that is, the expected number of unique genes found in each strain is non-zero). While the model is an insightful attack on the problem, we question the assumption that contingency genes are sampled in the population with equal probability. It is important to compare the existing model against a new model that does not rely on this assumption.

The Supragenome is represented here by a generative model that emits genomes according to a set of probabilistic rules. The supragenome contains *N *genes that are modeled as Bernoulli random variables with 'success' probabilities that correspond to the population frequency of each gene. A genome is generated by observing the Bernoulli variables: a gene is present if the corresponding trial is a success and otherwise absent. Each gene variable is assumed to be independent of all other genes. This assumption is sometimes violated in real *H. influenzae *genomes. For example, genomic islands are sets of genes that are not independent. However, we proceed with this assumption since it significantly reduces the complexity of the model and is reasonable in many cases.

The true population frequencies are, in general, unknown. Therefore, population frequencies are also treated in a probabilistic fashion. It is assumed that there are *K *discrete classes of genes. Each class *k *has an associated population frequency, μ_k_. All genes in class *k *will have population frequency μ_k_. Each of the *N *genes is assigned to a class according to a probability distribution given by the vector π, where π_k _is the probability that a gene is assigned to class *k*. Conceptually, π_k _is the percentage of genes in the supragenome that have population frequency μ_k_. The assignment of a gene to a class is independent of all other gene assignments.

**Figure 13:**
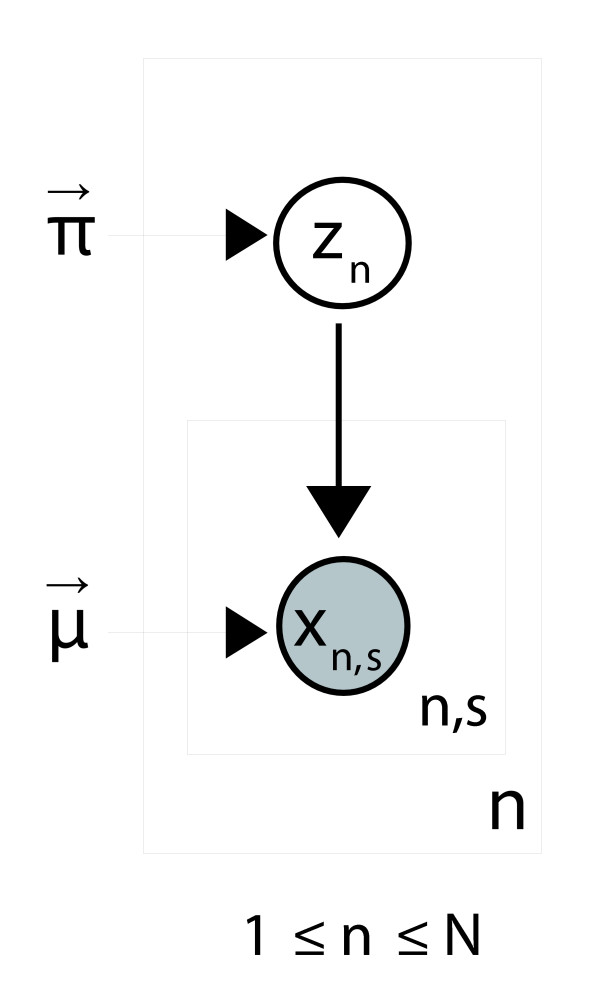
A plate diagram of the *H. influenza*e supragenome model. Each node in the diagram represents a random variable, and the arrows indicate dependence between the variables. Independent, identically distributed (IID) nodes appear in boxes with an index listed in the corner.

The complete model is depicted in plate notation in Figure 13. 'Z' is the hidden class variable in which *z*_n _corresponds to the class of gene *n*. 'X' is the observed gene variable, where *x*_n,s _corresponds to the presence or absence of gene *n *in strain *s*. The outer plate represents the supragenome, while the inner plate represents instances of specific genomes. The model requires 2 × *K *+ 2 parameters: *N*, *K*, a mixture coefficient π_k _for each class, and a Bernoulli probability μ_k _for each class. The number of gene classes, *K*, and their associated Bernoulli probabilities, μ_k_, are fixed in advance. Care must be taken to choose classes that represent low and high population frequencies. Seven classes were selected for this study (*K *= 7) with associated probabilities μ = <0.01, 0.1, 0.3, 0.5, 0.7, 0.9, 1.0>. The class with probability 1.00 represents 'core' genes that appear in all strains.

The remaining parameters, *N *and π_k_, are selected under a maximum likelihood scheme. Suppose that |*S*| genomes have been sequenced and a particular gene from class *k *was observed in *n *of the |*S*| strains. The probability of this observation is given by a binomial probability since this result is the sum of independent Bernoulli variables. As a function of π_k _and *N*, the probability is given by:

$$P\left(x=n|z=k,\mu_{k}\right)=\frac{\left|S\right|!}{n!\left(\left|S\right|-n\right)!}\mu_{k}^{n}{\left(1-\mu_{k}\right)}^{\left|S\right|-n}$$

However, we do not know the true gene class, so we must consider a mixture of binomial probabilities:

$$P\left(x=n|\vec{\pi },\vec{\mu }\right)=\sum_{k=1}^{K}P\left(x=n|z=k,\mu_{k}\right)\cdot P\left(z=k|\pi_{k}\right)=\sum_{k=1}^{K}\pi_{k}\frac{\left|S\right|!}{n!\left(\left|S\right|-n\right)!}\mu_{k}^{n}{\left(1-\mu_{k}\right)}^{\left|S\right|-n}$$

Now consider the complete set of genes. Let c = <*c*_0_, *c*_1_, ..., *c*_*S*_>, where *c*_n _is the number of genes observed that appear in exactly *n *of |*S*| strains. The probability of the total observation is given by a multinomial distribution:

$$\begin{array}{c} P\left(\vec{c}|N,\vec{\pi },\vec{\mu }\right)=\frac{N!}{c_{0}!c_{1}!\cdots c_{s}!}{\prod_{n=0}^{\left|S\right|}p\left(x=n|\vec{\pi },\vec{\mu }\right)}^{C_{n}}\\ =\frac{N!}{c_{0}!c_{1}!\cdots c_{s}!}{\prod_{n=0}^{\left|S\right|}\left(\sum_{k=1}^{K}\pi_{k}\frac{\left|S\right|!}{n!\left(\left|S\right|-n\right)!}\mu_{k}^{n}{\left(1-\mu_{k}\right)}^{\left|S\right|-n}\right)}^{C_{n}} \end{array}$$

The parameters *N *and π can be determined by maximizing the log-likelihood of the observation c:

$$\log P\left(\vec{c}|N,\vec{\pi },\vec{\mu }\right)=\log N!-\sum_{n=0}^{\left|S\right|}\log\left(c_{n}!\right)+\sum_{n=0}^{\left|S\right|}c_{n}\log\left(\sum_{k=1}^{K}\pi_{k}\frac{\left|S\right|!}{n!\left(\left|S\right|-n\right)!}\mu_{k}^{n}{\left(1-\mu_{k}\right)}^{\left|S\right|-n}\right)$$

The log-likelihood function was maximized by fixing *N *and maximizing with respect to π. The maximization was performed using the MATLAB function *fmincon *with the constraint:

$$\sum_{k=1}^{K}\pi_{k}=1$$

and requiring that the coefficients are between *0 *and *1*. The maximization was performed for values of *N *starting at the minimum possible value (the number of genes actually observed) to 6,000. The combination of *N *and π that maximized the overall log-likelihood was selected as the best parameter estimate.

### Supragenome modeling validation and results

**Figure 14:**
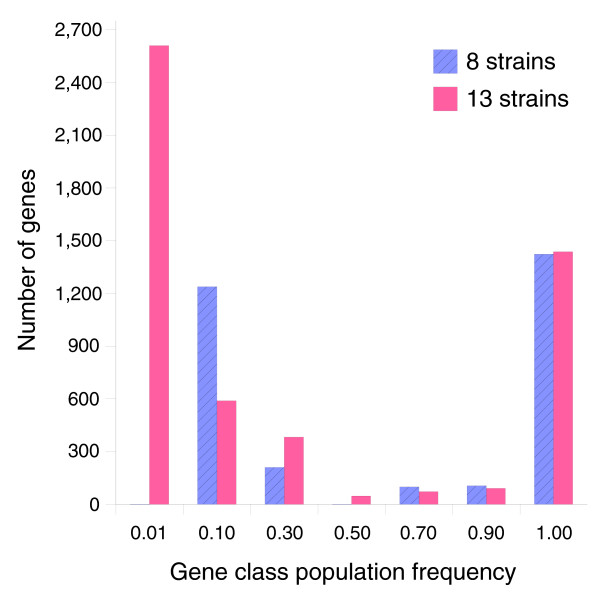
The distribution of genes among gene classes in the supragenome model trained on 8 or 13 strains. The only significant difference occurs in the rare gene categories with frequency 0.01 and 0.10. A small sample of eight strains is not expected to generate accurate predictions for these categories.

The model was validated by training the supragenome parameters using only the first 8 sequenced genomes and comparing the predictions with the observed results for 13 strains. The maximum likelihood number of genes was 3,078. Of these genes, 1,423 are core genes, 417 are contingency genes with population frequency >0.1, and 1,238 are contingency genes with 0.1 population frequency. No genes were predicted in the 0.01 population frequency class. Predictions for the 0.01 class may be inaccurate due to the small sample of 8 genomes. The 1/100 maximum likelihood confidence interval for total genes ranged from 2,975 to 3,681. Figure 14 shows the distribution of the genes among the seven classes.

Figure 5 compares model predictions based on 8 strains to actual observations of core genes (shared among the first N strains) and total genes found after sequencing the 9th through 13th strains. In both cases the model predictions follow the observed trends. Figure 6 compares predictions to observations of the number of new genes found in the Nth sequenced strain. Again the model predictions follow the observed trend. Figure 2 compares the best-fit gene distribution (based on 8 strain models) to the observed distribution of genes found in exactly N of 13 strains. Overall, the predicted trends follow the observed distribution; however, the predictions were too low for genes seen in 1 of 13 strains, and too high for genes seen in 2 of 13 strains. This bias may be due to the small sample size (eight strains) used to train the supragenome model. Predictions for genes seen in four to seven strains were also somewhat lower than observed.

The supragenome model predicted an average of 1,776 genes per strain with a standard deviation of 14 genes. Of the 13 strains, the average number of genes was 1,793 with a standard deviation of 62 genes. The model predicted an average of 373 different genes when comparing any two strains with a standard deviation of 17 genes. Among the 13 sequenced strains, the average was 395 with a standard deviation of 91 genes. In both cases the model predication for average was reasonable, while the standard deviation was underestimated by about four-fold. This suggests that the assumptions used for the supragenome model may omit important sources of variation. Genomic islands and other genes that appear together in the genome likely contribute to the total variance.

**Figure 15:**
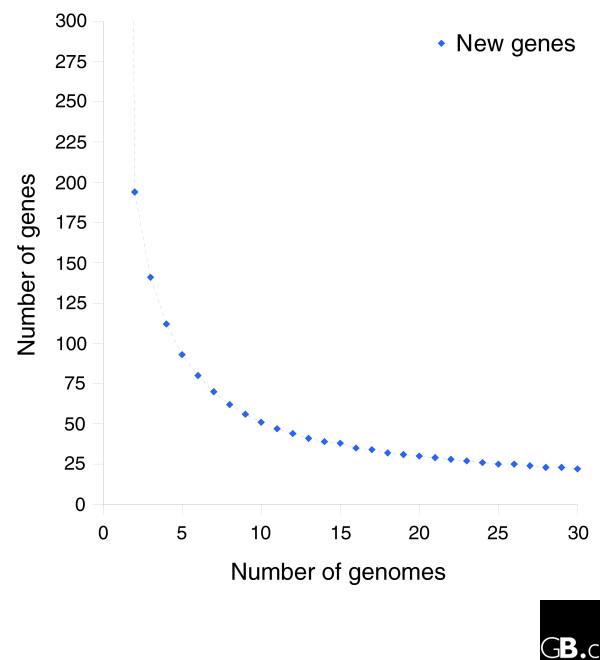
A theoretical plot of the number of new genes expected to be found in the Nth genome for future *H. influenzae *sequencing projects. The plot was generated using strains isolated in North America, and the extrapolation may not hold for isolates from other geographic locales if some distributed genes are geographically isolated. The model predicts that the number of new genes found in a strain will diminish 20 after sequencing 30 strains, and the number will trend toward 0 as the number of sequences becomes large.

**Figure 16:**
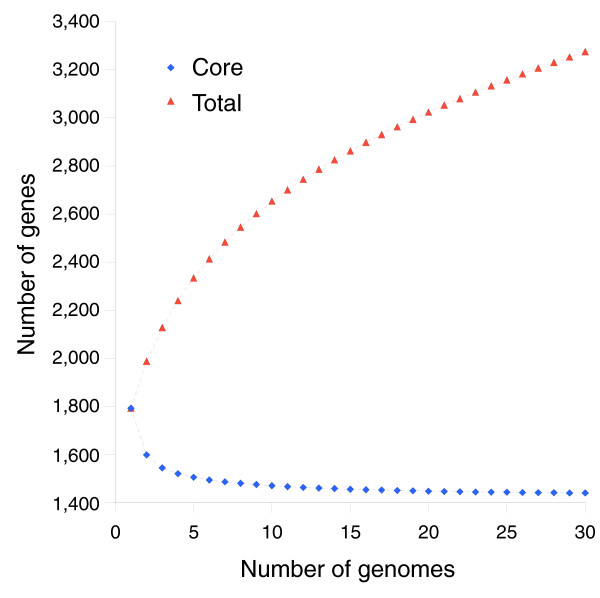
A theoretical plot of the number of total genes and core genes expected among N sequenced *H. influenzae *genomes for future sequencing projects. The extrapolation may not hold for strains isolated outside of North America since the plot was constructed using only North American isolates. The number of core genes approaches an asymptote, which reflects a common set of genes present in all natural isolates.

Altogether, the above results show that the supragenome model generates reasonable predictions for the average properties of the supragenome. To obtain improved predictions, the model was re-trained on all 13 strains. The supragenome class distribution for the extended model is shown in Figure 14. The results are similar to the model trained on 8 strains, except that the class with population frequency 0.01 is now predicted to contain 2,609 genes, while the 0.10 frequency class was reduced in size to 590 genes. This large change is due to improved resolution of rare genes in the 13 strain training set. The model now predicts 5,230 genes, with a 1/100 likelihood interval ranging from 4,425 to 6,052 (Table 9). Nearly all of the increase over the eight strain model is due to the class of rarest genes. Of these genes, 1,437 are core genes, 594 are contingency genes with population frequency >0.1, and 3,199 genes are rare contingency genes with population frequency <0.1. Figures 15 and 16 show the prediction trends for total, core, and new genes observed after sequencing N strains (up to 30 strains).

**Table 9:** Maximum likelyhood estimate for size of supragenome and 1/100 likelihood intervals based on 8 and 13 strain training sets

	Training set	
	
	8 strains	13 strains
Lower bound	2,975	4,425
MLE	3,078	5,229
Upper bound	3,681	6,052

## Discussion

Comparative genomic analyses were performed on 13 *H. influenzae *strains, 12 clinical isolates and Rd, an acapsular strain derived from a serotype d strain that is not typically associated with disease. The results of these studies demonstrated great genic diversity among the strains on average. This genic diversity is visualized by a dendrogram constructed from the genic differences among strains (Figure 4). A typical pair of strains varied by nearly 400 genes. A phylogeny constructed from MLST housekeeping genes also demonstrates a high degree of allelic diversity. However, the topologies of the MLST and genic trees differ significantly. This indicates that the genic sharing of non-core genes among strains is not always related to the phylogenetic relationships inferred from housekeeping genes. Rd was not an outlier in either tree, suggesting that encapsulated strains share the same supragenome. This reinforces previous research that arrived at the same conclusion using other methods [11]. Cluster analysis revealed nearly 2,800 distinct genes among these 13 strains, while modeling predicts that the species-level supragenome will contain 5,000 or more genes and require the analysis of several hundred strains to be complete. A supragenome containing approximately 5,000 genes would possess nearly three times the number of genes observed in any single strain.

Slightly over half (1,437) of the gene clusters identified are predicted to constitute a necessary set of core genes. The non-core genes in each strain (356 on average) are composed of distributed genes (present in more than one strain, but not all strains) and unique genes that are not represented in any other *H. influenzae *strains. Genes in the core genome are more likely to display typical *H. influenzae *codon usage patterns and are rarely homologous to phage-related genes. In contrast, the distributed genes and unique genes are more likely to display atypical codon usage patterns for *H. influenzae *and are more likely to share homology with phage and other bacterial species, but still the majority of these non-core genes possess codon usage statistics similar to core genes. In fact, out of a total of 736 distributed genes observed among the 13 strains, less than 15% displayed any significant phage homology. Hence, the core genome is wholly specific to *H. influenzae*, while non-core *H. influenzae*-specific genes are likely mixed with genes of foreign origin. The subset of contingency genes with typical codon usage patterns and without phage homology will be important candidates for functional studies.

Among the 13 strains examined, 539 unique genes were identified. Our model predicts that most of these 'unique' genes are derived from a pool of approximately 3,000+ low frequency genes. Of these, 25% demonstrate sequence homology to phage genes. The codon usage of these genes is often typical, but more likely than core and distributed genes to diverge from *H. influenzae *patterns. The origin and importance of the remaining 75% of the unique genes is unclear. Since these genes have not been enriched in the population by positive selection, it is uncertain whether these genes correspond to a functional role in *H. influenzae*; however, previous studies have demonstrated that 100% of the unique genes examined are expressed as RNA transcripts [11]. It is possible that high levels of horizontal gene transfer between organisms in the *H. influenzae *environmental niche results in a number of uncommon genes stranded at any particular time point in any given strain. Evolutionary processes will remove genes not providing a selective advantage over time, but this may be a slow process in comparison to the acquisition of genes by horizontal gene transfer. In other words, evolutionary processes may be unable to 'empty the trash' quickly enough to eliminate all non-useful genes simultaneously. The energetics penalty imposed by a single non-useful gene is likely to be small, yet the cumulative effect of many such genes could be significant. A balance between the rate of gene acquisition by HGT and negative selection due to energetics is a likely mechanism contributing to the maintenance of the overall genome size. It is also possible that many of these unique genes are recent functional additions to the NTHi supragenome, but have not yet had time to become widely dispersed. There are a number of environmental factors that have been profoundly altered over the past half century that could account for this, including widespread antibiotic usage and high density human daycare for infants, which results in much higher rates of polymicrobial respiratory infections.

Our clustering methods were designed to minimize bias due to frame shifts and assembly gaps. Nonetheless, the number of clusters identified with these methods may contain some such bias. Sequencing errors may induce frame shifts that split a gene into two fragments. Clusters of orthologous genes (COGs) is a common method for identifying gene orthologs across a wide range of species. The COG method is able to discriminate between closely related paralogs by using only bi-direction best homology matches (BBH) while constructing clusters [28,29]. Since the COG method requires BBH, if a split ORF is present, only one of the fragments will cluster with the full length gene. This results in orphaned 'genes', which inflate the number of gene clusters observed. To resolve this issue, we implemented a less restrictive clustering algorithm that uses uni-direction homology matches above a minimum sequence identity and a minimum fraction of the length of the shorter gene. Furthermore, six-frame gapped translations are used during homology searches to minimize the impact of sequencing errors. The disadvantage of our approach is that paralogs may cluster together if the sequence identity is above the threshold. However, since the genes under consideration are from the same species, the orthologs are expected to be highly homologous in comparison to paralogs.

Accurate clustering depends on careful selection of parameters. We started with the observation that sequence identity among orthologs is higher, on average, than among paralogs. To find the best parameters, we examined a plot of the number of clusters as a function of the parameters (Figure 1). In the case of the identity parameter, a low threshold will cause all paralogs to group together, which results in a small number of clusters. As the threshold increases, the number of clusters increases as paralogs are segregated into distinct ortholog classes. When the threshold passes the peak of the paralog distribution, the rate at which clusters split is reduced. But, as the threshold increases further, ortholog clusters begin to split, and the number of clusters increases more rapidly. At 100% identity threshold, all but the most highly conserved orthologous clusters have been split apart. Figure 1 reveals an inflection point in the region between 60% and 70% identity where the slope is decreasing and then starts to increase. The inflection point suggests that an identity threshold of 70% defines the best partition between paralogs and orthologs. Analogous reasoning was employed in determining the match length threshold.

Another bias may be introduced by the use of unfinished genomes in this study. Despite assembly gaps, the likelihood that an entire gene is missing from the sequence is low due to the high coverage (>25×, on average) generated by the 454 sequencing method. Lander-Waterman statistics predict that more than 99.9% of each genome was sequenced. Most gaps are due, therefore, not to missing sequences but rather the difficulty of assembling repeat sequences. On average, 1,769 gene clusters were found per completed genome versus 1,804 for unfinished genomes. This difference is most likely due to real genomic differences as supported by metabolomic studies (data not shown), but in the worst case the difference is an upper bound on the error.

An important consequence of our supragenome model is that the observed diversity among the *H. influenzae *strains can be adequately explained by a finite model. This contrasts with conclusions drawn from models built for the pathogen *S. agalactiae *[27]. Our study does not contradict previous analysis, but emphasizes that conclusions are dependent on modeling assumptions and the species in question. While it is tempting to assume the supragenome of a naturally transformable species draws from the nearly infinite pool of genomic diversity found in nature, several factors make it likely the pool is quite restricted. The first barrier is environment. In the case of *H. influenzae*, only species that co-habitate in the human respiratory mucosa are available for genetic exchange on a regular basis. The second barrier is a set of mechanistic restrictions built into the transformation system. Uptake of DNA is enriched by the presence of uptake signal sequences, which are commonly present in *H. influenzae *genomic DNA but are not common in other species [30,31]. After uptake, sequence homology is necessary for efficient incorporation of DNA into the chromosome via homologous recombination. Consequently, most HGT events among *H. influenzae *are expected to derive from its own population and to a lesser degree from genetically similar species residing in the same environmental niche. Our model predicts a pool of rare genes in the range of approximately 2,700 genes - this may reflect the number of genes available to the organism from genetically similar species living in the same environmental niche. This reasoning does not exclude the potential importance of rare HGT events between distantly related species on an evolutionary time-scale.

While a global analysis of the supragenome is important, the ultimate goal is an understanding of the phenotypes associated with individual genes and combinations of genes and how these contribute to the process of disease. The sequence data obtained from this study will serve as a valuable tool in this endeavor. The collection of genes identified here will be used to construct a supragenome hybridization (SGH) chip, analogous to a eukaryotic comparative genomic hybridization (CGH) chip. The SGH chip will be used as a low-cost genome screening tool for a large number of clinical NTHi isolates for which disease phenotype data are available. The resultant data will be used to generate gene association studies for the identification of genes and gene combinations that contribute to various disease processes.

## Conclusion

The results reported herein provide evidence of a significant population-based supragenome among clinical strains of the NTHi, as well as substantive support for the DGH. The observation that, on average, every clinical strain varies from every other clinical strain by the presence or absence of over 300 genetic loci is highly suggestive that there is enormous heterogeneity among NTHi strains with respect to their pathogenic potential. These findings point the way toward future studies in which statistical genetic approaches could be brought to bear on the identification of associations between particular sets of genes within the supragenome, and the discrete clinical disease phenotypes of the individual strains. As these genic association data become available, it should be possible to develop next-generation molecular diagnostics to help with the prediction of disease treatment and outcome based upon the particular infecting population.

## Materials and methods

### DNA sequencing

Complete or nearly complete genomic sequences of 11 unique clinical strains of *H. influenzae *were generated and used in comparative genomic analyses with the two published NTHi genomes [32,33] in the development of a supragenome model. Genomic sequence of nine clinically isolated NTHi strains was generated at The Center for Genomic Sciences by the 454 Life Sciences GS-20 sequencer using standard protocols [25]. Strains were sequenced to a depth of 16×, or greater, and assembled *de novo *by the 454 Newbler assembler to 81 contigs, on average. Lander-Waterman statistics predict that greater than 99.9% of each genome was sequenced. Regions of duplicated sequence caused most of the assembly gaps. Informal comparison between high-quality Sanger reads and 454 data suggest an error rate of less than 1 in 1,000 bases. Most base call errors are single base insertions or deletions in homonucleotide repeats that can result in frame-shift artifacts. The other two clinical NTHi isolates (R2846 and R2866) included in the comparison were sequenced at the University of Washington Genome Center (Alice Erwin, personal communication). The complete genomic sequences of *H. influenzae *strain Rd KW20 and 86-028NP and the incomplete sequences of strains R2846 and R2866 were accessed through the Microbial Genomes Database of NCBI.

### Accession numbers

The most recent versions of the genome assemblies were deposited with GenBank, with the following accession numbers for the indicated strains: CP000671 (CGSHiEE); CP000672 (CGSHiGG); AAZD00000000 (CGSHi22121); AAZJ00000000 (CGSHi22421); AAZF00000000 (CGSHi3655); AAZG00000000 (CGSHiAA); AAZH00000000 (CGSHiHH); AAZI00000000 (CGSHiII); and AAZE00000000 (CGSHiR3021).

### Partial genomic assembly of 454-based genomic sequences

The 454-assembled PittEE strain genomic contigs were scaffolded against all four of the completed *H. influenzae *genomes using Nucmer [34], which indicated the greatest similarity to strain 86-028NP. Using a maximum parsimony approach, the PittEE genome was reduced to 12 contigs by a combination of: sequencing PCR amplicons targeted to fill gaps between neighboring contigs, as inferred by the scaffolding; and sequencing a 4 kb clone library and searching for clones that spanned gaps in the 454 sequence. Gap closure experiments were designed by a custom Perl script, and PCR primers were designed by Primer3 [35]. Similarly, PittAA was reduced to 47 contigs by sequencing of PCR amplicons generated following scaffolding. Clones and PCR amplicons were assembled along with 454 contigs by a modified Phred-Phrap-Consed pipeline where 454 contigs were converted to PHD format files and input to Phrap as long reads [36-39].

### Gene identification

Coding sequences for all 13 strains, including those previously annotated, were identified by the AMIgene microbial gene finder adjusted to low-GC parameters and trained on the Rd KW20 genome [40]. AMIgene builds three Markov models to identify coding sequences with different codon usage statistics. This provides increased sensitivity for genes of possible foreign origin. Prior to gene calling, all contigs were artificially stitched together using a linker (NNNNNCATTCCATTCATTAATTAATTAATGAATGAATGNNNNN) that provided start and stop codons in all six reading frames, permitting the identification of genes that extend past the ends of a contig [27].

### Gene clustering

Each pair of genes was examined for protein homology by alignment of six-frame nucleotide translations to predicted protein sequences. Alignments were generated by tfasty34, part of the Fasta v3.4 package [41]. Six-frame translations were employed to minimize the impact of frame-shift artifacts. Each gene was also aligned against the full nucleotide sequence of the 13 genomes by fasta34 (also part of the Fasta package): Fasta34 parameters, fasta34 -H -E 1 -m 9 -n -Q -d 0; Tfasty34 parameters, fasty34 -H -E 1 -m 9 -p -Q -d 0. Genes were clustered based on homology using a single-linkage algorithm. A link was defined by a significant tfasty match between genes that exceeded an identity threshold of 70% and covered at least 70% of the shorter gene (a detailed discussion of parameter selection is found in the supplementary materials at [42]). The asymmetric length criterion was chosen to insure that fragmented genes would cluster with the full length version of the gene. A side-effect of this criterion is that multi-domain proteins may fuse with proteins that are composed of a subset of those domains. Significant fasta matches between genes and genomic sequence were used to identify sequence conservation between a gene cluster and a strain. In the event of a significant match (70% identity/70% length), the matching genome was considered to possess the gene cluster for purposes of quantifying the number of strains that contain the gene cluster. See supplementary materials for a comparison of our clustering methods and the COG method [42].

Multi-alignments were generated for each cluster using poa (partial order alignment) in order to visually and computationally verify the integrity of the clusters [43]. If the multi-alignment of a cluster was less than 120 bp in length, the cluster was filtered as a likely false-positive gene. Finally, an attempt was made to split false clusters formed by multi-domain proteins by searching for point of partition in the multi-alignment that divided the majority of genes into two non-overlapping sets. The algorithm was implemented using a custom Perl script.

### Phylogenetic tree building

Two types of dendrograms were generated and compared. A gene possession-based phylogenetic tree of the 13 NTHi strains was constructed by defining the distance between a pair of genomes *i *and *k *to be:

$$\sum_{n}|g_{n,i}-g_{n,k}|$$

where *g*_*n*,*i *_= 1 if gene *n *is present in strain *i *and 0 otherwise. The strains were clustered based on the distance metric by the unweighted group average method implemented in the Phylip package [44-46]. A tree was also generated using sequence alignments of seven housekeeping genes used in multi-locus sequence typing [47]. The tree was constructed using the maximum likelihood method implemented in fastDNAml as part of the Phylip package [48,49].

### Whole genome alignment

Whole genome alignments were generated by Nucmer and visualized by Mummerplot [34]. MUMmer parameters were set to -maxmatch -l 16 -o. The order of PittEE contigs was inferred from optical restriction fragment maps generated by Opgen (Madison, WI, USA) [50]. Whole genome alignments were not built for most strains since the ordering of the contigs was not determined.

### Insertion-deletion analysis

Inserted and deleted genomic sequence, in comparison to the Rd KW20 genome, was identified by maximal sequence matching performed by Nucmer [34] with the settings -maxmatch -l 16 -o. Non-matching sequence was identified and quantified by a custom Perl script.

### Multistrain local sequence alignments

Multistrain local sequence alignments against reference sequences (86-028NP or Rd KW20) were generated using BLASTn [51] by querying the reference sequence against a database containing the genomic sequence of all 13 strains. Alignments were then visualized using BioPerl scripts. By the nature of this alignment procedure, sequence that is present only in non-reference strains is not visualized. Gene annotations for reference strains were obtained from GenBank.

### Phage homology analysis

Phage derived gene clusters were identified by selecting a representative sequence from each gene cluster to use as a BLASTx query against the NCBI NR (non-redundant) protein database. GenBank records of the top ten significant protein matches with e-value >1e-8 were queried for the keyword 'phage'. If the keyword was identified among the matches, the gene cluster was flagged as 'phage derived'.

### Codon usage analysis

The codon usage of a representative sequence from each cluster was analyzed by CodeSquare using Rd KW20 mean codon usage as a reference [26]. The epsilon statistic reported by CodeSquare was normalized for ORF length dependence using a best-fit power function for the mean and variance (as a function of length). Gene clusters were divided into three categories: core (gene found in all 13 strains), contingency (2-12 strains), and unique (1 strain). To minimize length bias, codon usage analyses were limited to genes with lengths between 200 and 300 amino acids. Significant differences in the median epsilon statistic were calculated using the non-parametric Mann-Whitney U test.
